# Novel CSR & novel coronavirus: corporate social responsibility inside the frame of coronavirus pandemic in Greece

**DOI:** 10.1186/s40991-021-00065-7

**Published:** 2021-08-06

**Authors:** Ioannis Panagiotopoulos

**Affiliations:** 1 Department of Business Administration, University of the Aegean, 8 Michalon Str, 82132 Chios, Greece; 2 Co- Founder of School Life Museum of Thisvi, Voeotia, Greece; 3 Attica, Greece

**Keywords:** Corporate Social Responsibility, Stakeholders, Economic & Health Crisis

## Abstract

Corporate Social Responsibility (CSR) becomes popular as big international firms gain more power than states and global issues engender concerns to people from all over the world. The pandemic of novel coronavirus is a current issue of global concern that threats humanity and global economy since the end of 2019. A lot of firms have announced urgent actions to support their employees and the local communities. The present study aims to examine whether the CSR activities of firms due to the pandemic could be categorized either as strategical or tactical CSR. The researcher recognizes the formation of a new kind of CSR called critical CSR as a hybrid between tactical and strategical CSR sharing characteristics from both. The examination of the case of Greece during the pandemic has provided a variety of examples of CSR activities from big Greek international firms that have been processed to support the validity of the reasoning. Finally, the paper delineates this new universal form of CSR born under the critical circumstances of the pandemic and the ensuing economic recession. That fact proves that this crisis could be transformed into a chance for corporations to realize their social role and improve their CSR footprint with the learnings of this pandemic by underlining possible advantages of these urgent CSR actions that could be incorporated into the usual CSR policy of the firms.

## Introduction

Corporate Social Responsibility (CSR) is the concept that calls corporations to consider social and environmental concerns to their decision- making process (European Commission, 2001). CSR suggests a live connection among a corporation with sustainability principles meaning an equilibrium among a) economy, b) society and c) natural environment (Elkington, 1999) using the prism of Ethos (Carroll, 1979, 2016; Frederick, 1998) with its ancient Greek meaning. Ethos or ethics in modern English refers to a moral character who can distinguish good and bad, right and wrong, and continues doing the right, good, ethical thing (Eisenstein, 2020).

CSR concerns a planning of activities that are beyond the simple compliance with the law and profit making. However, specialists in designing CSR policies are stimulated by the existed legal frame and could be inspired from statutes. Big international corporations should design a CSR strategy that expresses their sustainable principles inside the frame of globalization adjusted in the everyday activities of them under the usual conditions of our world.

However, currently humanity faces an unprecedented and unusual circumstance even though we have faced three coronaviruses during twenty-first century having as main effect respiratory disease outbreaks and finally death. SARS came up in 2002 in China and MERS in 2012 in KSA. COVID-19 was first reported in China in 2019 and up to end of May 2020 had been spread all over the world except Antarctica. Both SARS and MERS presented higher fatality rates than COVID-19 but the last has much easier dispersion that leads to greater number of cases and deaths. World Health Organization (WHO) declared COVID-19 as a pandemic on 11^th^ of March 2020 (Hewings- Martin, 2020).

Most of the countries have applied several types of social distancing and lock- down to their societies and markets respectively aimed at limiting person-to-person contact. Although coronavirus is a health enemy, it hits economy equally strongly since this general lock-down and social distancing affects economy with variable ways. We could recognize as side effects of coronavirus the decrease of production in primary sector like agriculture and the secondary sector like car industry since social distancing affects the occupation of workforce. The same applies for the tertiary sector with the example of the crisis in transportations, tourism, and aviation due to the same reasons. The pandemic of COVID-19 has symmetrical characteristics because it hits evenly: a) the demand for goods and services and b) the demand and the offer of them.

There is an extensive debate publicly by scientists, politicians, economists, etc. regarding the abovementioned side-effects of the pandemic and the measures of protection like social distancing. Part of public opinion and specialists support that these strict protection measurements have increased the side-effects of the pandemic especially in economic sector (Ioannidis, 2020) where we face the biggest recession of the last 100 years with decrease in global Gross Domestic Product (GDP) of 3.5% (and in Greece of 8.2%) (Stournaras, 2021). This consideration has directed some countries like USA, UK, and Sweden to be more reluctant to impose strict measures of social distancing and lock-down. However, even without these protection measures seems to be inevitable for global and national economies to avoid recession when there is such intense dispersion of such a deadly virus. Indicatively, there were recorded 119 M cases and 2.6 M cumulative deaths exactly after a year since declaration of the pandemic (11 March 2021) and approximately 180 M cases and 4 M patients who passed away up to end of June 2021 (John Hopkins University, 2020).

The treatment of this pandemic occupies the plurality of medicine departments in universities and pharmaceutical industry which are looking for the proper treatment and the vaccines which could enhance human defense against coronavirus. However, especially the first semester of 2020 the actions were clearly organized in country level even in the case of coalition of states like EU. Countries failed to develop a closed cooperation among them regarding initially the health issue and afterwards the financial issue due to the pandemic and they reacted competitively rather than cooperatively. It seems that we did not learn from the two prior epidemics of coronavirus and were ill-prepared to deal with the challenges the COVID-19 epidemic has posed (Peeri et al., 2020). Finally, big countries and coalitions of states like USA and EU respectively decided to invest in common in medical researches for vaccines and to purchase them for all member-states. Moreover, both have announced plans for financial recovery for the post-COVID-19 era of several trillions through special laws and programs like the Coronavirus Aid, Relief, and Economic Security (CARES) Act (Gravelle & Marples, 2021) and the NextGenerationEU program (European Commission. Directorate General for the Budget., 2021) respectively.

Corporations must face the threat of coronavirus and the forthcoming depression by adjusting their operation under the new pandemic conditions keeping their personnel, customers, suppliers, and rest stakeholders safe, protecting their profitability on the same time. This complex problem consists an urgent call for firms to express their social sensitivity and implement urgent CSR programs for their employees and society.

## Manifest

In general, corporations implement either strategic or tactical CSR policies so far. Strategic CSR is the incorporation of an holistic CSR perspective within a firm’s strategic planning and core operations so that the firm is managed in the interests of a broad set of stakeholders to achieve maximum economic and social value over the medium to long term. Tactical CSR refers to short term management decisions about CSR programs of limited resources and minimal impact to firm’s core operations (Werther & Chandler, 2006). Thus, it is expected that firms will design urgent CSR programs that could be categorized either as strategic or tactical CSR. Having seen a plethora of CSR actions by international firms due to COVID- 19 could we consider the urgent CSR activities of firms due to the pandemic as a usual CSR activity? If yes, we could categorize them either as tactical or strategical CSR activities based on their special characteristics. Moreover, are there lessons to learn for corporations from their reaction to the pandemic? Could corporations borrow useful elements from their urgent CSR activities and incorporate them into their usual CSR policy?

The researcher manifests that this urgent CSR activity of firms due to the pandemic belongs neither in tactical nor strategical CSR policy of them. It is about a totally new kind of CSR, a hybrid between tactical and strategical CSR sharing characteristics from both types of CSR. This new kind of CSR called critical CSR by the researcher since it is inevitable and crucial under these intense pandemic conditions. It consists the result of firms’ reaction and response to a totally new phenomenon, a worldwide pandemic that causes a global recession too. Critical CSR is crucial since really helps employees, society, states, and companies themselves to resist this pandemic. This new, universal type of CSR is critical for firms since wherever they miss to take initiative to confront this pandemic the states will intervene to cover the gap. Furthermore, critical CSR is inevitable having intense ontological nature because public health and global economy are in danger and corporations should defend their human resources and their market. Without these two elements, the financial game cannot exist. Finally, critical CSR enhances and enriches CSR concept. Several elements of critical CSR could be used aftermath this pandemic to improve the usual CSR policy of firms.

## Methodology

The researcher uses the method of reductio ad absurdum (proof by contradiction) to argue for his statement. Whether the new CSR activities of firms during the pandemic are considered as part of usual CSR, they could be categorized either as tactical or strategical CSR. This entails that they should fulfill either the relevant strategical or tactical criteria. A possible weakness to be described either as tactical or strategical based on their special characteristics could direct us to consider the possibility of birth of a new kind of CSR under the current totally turbulent conditions.

The present research is based on an unobtrusive research, a qualitative and comparative content analysis (Babbie, 2011) in the field of CSR programs implemented during the period of the pandemic by Greek international companies in Greece. The unit of analysis is the CSR policy of a firm and the units of observations are the firms under examination. The firms are selected based on four criteria:

- the importance of the urgent CSR program based on amount of money invested or the criticality of the parameter where the intervention occurs, meaning CSR programs that targets with high precision to the pandemic are preferred like enhancement of medical capabilities rather than a donation for poverty for example,
- the level of publicity of the urgent CSR program, companies which have well communicated their urgent CSR programs are preferred as indication of a deeper engagement with CSR examining press releases, mentions in corporate websites, etc.,
- the widest representation of the biggest part of Greek economy, one company per sector of Greek economy is considered enough, and 15 sectors have been examined in total,
- the diversity of actions undertaken is preferable to highlight the plurality of CSR actions and innovative ideas under these unprecedented circumstances, meaning that prototype CSR programs of lower value could be selected instead of a series of similar CSR programs of higher financial value.

The presented herein cases concern Greek firms most of them with international presence that are protagonists in their sectors, and they could be consider big size firms with more than two hundred employees. By the term “Greek” we mean companies with Greek origin which maintain part of their production and activities in Greece as well as their physical headquarters. By the term “international” we mean companies with presence inside and outside Greece with a significant percentage of exports, branches, or affiliate companies abroad. The researcher examines several examples of CSR actions against coronavirus from all the central pillars of Greek economy. A data base with 15 firms has been created covering fifteenth of the most important sectors of Greek economy (*Greece’s Economic Performance and Prospects*, 2001; Stournaras, 2021) from banking up to tourism and maritime.

The information is retrieved through research in specialized business and economic newspapers in internet, TV programs, corporate websites, and published CSR reports. Furthermore, literature review has been conducted regarding the modern CSR developments and the conclusions aggregated regarding the consequences of past economic crises in CSR programs of international firms. The researcher tackles the opportunity to make comparisons among current health and financial crisis with the previous financial crisis of 2007–2009, to foresee the implications in CSR field and recognize useful good practices and lessons for the next day of CSR.

## Theoretical considerations

The second half of twentieth century there was a significant development in the theoretical complex of CSR. Several definitions and different ways for categorization based on discrete aspects offer various ways to examine CSR policy of firms. Fundamental elements of CSR theory will be presented in this paragraph since they are going to be used aftermath for better understanding critical CSR.

This global crisis is both medical and financial and consists the next economic shock for global economy after 2007–2009 recession. So, it is very interesting to see whether CSR declines, remains stable or even has been enhanced during this turbulent period for global health and international economy. During a financial crisis, the CSR strategy of an organization is challenged. The reactions of a firm during a financial turnover regarding its already designed CSR strategy could be used as an indicator of CSR embeddedness into corporate strategy (Fehre & Weber, 2016). Big part of public opinion assumes reasonably that CSR policy is threaten during a depression and finally managers tend to abandon it during a downturn period. A study by Bansal et al., (2015) pays deep attention to the distinction between strategic and tactical CSR. It recognizes that all CSR activities are bent during recessionary periods. However, it is supported that strategic CSR programs are more resilient than tactical CSR programs since companies prefer to pull back on the tactical level. A company with good Corporate Financial Performance (CFP) prefers to maintain strategic CSR programs and reduce its exposure to tactical CSR programs during a recession.

Proportionally to the concept of CFP there is Corporate Social Performance (CSP). It is about the real endeavors of a company to implement its CSR policy. A lot of definitions have been given by specialists and one of the most completed is coming from Wood (1991) as an arrangement of CSR principles, processes of social responsiveness, related policies and programs. The concept of CSP is coming as answer to the question of how much a firm has embraced CSR principles and how intense are firm’s efforts to implement its designed CSR policy.

Tactical CSR is directed to assist stakeholder relationships in the short- term. Tactical CSR programs often require less resources, do not imply significant changes in firms’ structure and operation. Thus, tactical CSR programs could be applied promptly, and they are usually reversible. On the contrary, strategic CSR includes bigger commitments in resources, requires medium to long horizons, affects firms’ operation and structure. Therefore, strategic CSR programs are more idiosyncratic to the firm, difficult to be copied by competition and reverse whether conditions change (Bansal et al., 2015).

From a different point of view, CSR is categorized depending on motives and actions held into 3 types: a) ethical CSR, b) altruistic CSR and c) strategic CSR. Ethical CSR in brief focuses on firm’s responsibility to avoid any detrimental action against society and environment (Carroll, 1991). Altruistic CSR is going one step further by indicating exactly what ethical CSR does plus firm’s contribution to current issues of society like carrying out philanthropy even by having a negative financial impact on them (Lantos, 2011). Strategic CSR goes one step further by recognizing the satisfaction of firm’s stakeholders as a way to fulfil firm’s traditional goals for profitability (Jones, 1995; Mc Williams & Siegel, 2011).

Accordingly, there is a three- levels classification of companies as: a) reactive, b) responsive and c) interactive (Preston & Post, 2013; Sethi, 1979) based on the nature of their CSP. The compliance of firm’s actions with the laws refers to ethical CSR and a reactive company. Proportionally, firms which care for societal issues that are either directly or indirectly interwoven with their core activities plan altruistic CSR policies and belong to responsive type. Last, an interactive firm goes deeper applying strategic CSR and having a sound CSP. Moreover, a firm’s environment could be categorized in internal and external environment. The internal environment consists mainly of its employees and the external environment includes its customers, suppliers, partners, etc. and society in general (Carroll, 1979; Cornelius et al., 2008; Werther & Chandler, 2006).

## Context

The current study focuses on the case of Greece during the battle against coronavirus. Greece has started taken precautionary actions against the pandemic since February 2020 when it had less than 100 cases. The dramatic situation in neighboring Italy and the awareness of the insolvency of the National Health System due to limited number of Intensive Care Units (ICU) were the determining factors for the immediate activation of precautionary measures.

The recent depression in the country that kept more than ten years (2009- 2019) had left Greek hospitals with open wounds, lack of funding and personnel. Greece in the beginning of the pandemic had available 540 ICUs instead of a theoretical ideal number of 3.500 and a European average of 1250. The 540 critical care bed puts Greece’s capacity in the bottom of Europe because this fact equals with less than 6 ICUs per 100,000 people when the European average is 11.5. This means that Greece should double the number of beds in order to reach the European average (Rhodes et al., 2012). This has operated as a strong alarm from Greek government and as a key performance indicator for country’s efforts.

Taking into consideration the available data up to the first semester of 2021, Greece has implemented a very good plan against coronavirus. Its performance has led it among the safest countries in Europe during this pandemic. Very relevant with the CSR activities of firms along with the contribution of charity organizations is the fact that Greece has managed in a year (March 2020- March 2021) to almost double the available ICU beds in the country from 540 to 1000 (951 ICU beds) approaching the European average. These two abovementioned facts of country’ highest ranking among the safest countries and the vertical improvement in the medical infrastructures indicates that state, doctors, civil protection, simple citizens, charity organizations, and companies through their urgent CSR programs have well managed this crisis so far.

## Findings

Several urgent CSR actions have been taken by big international Greek corporations that are beyond the laws for the confrontation of pandemic aiming to protect their employees and society in general. All the examined companies have already been familiar with CSR principles, plan annual CSR policies and some of them publish sustainability reports. It is useful to examine and compare these urgent actions in depth and detect the axes they are developed on.

These urgent actions involve both the external and the internal environment of the selected firms. Companies have tried to protect their employees by applying their flexibility for remote work, less business trips, less physical meetings, and increased usage of Information Technology (IT) capabilities. They have offered training to their employees about protection measures against COVID-19. On the other side, companies have tried to support their external environment against coronavirus. They have offered money, medical equipment and services to hospitals, research foundations and civil protection organizations, etc. Table 1 presents this aforementioned information and the existence or no of an established CSR policy and a published periodically CSR report in its last column.

**Table 1:** Representative CSR activities by Greek firms during the pandemic

**SECTOR OF MARKET**	**COMPANY**	**URGENT CSR FOR SOCIETY/ EXTERNAL ENVIRONMENT**	**URGENT CSR FOR EMPLOYEES/ INTERNAL ENVIRONMENT**	**EXISTENCE OF CSR POLICY/ REPORT**
SHIPPING- MARITIME	KYKLADES MARITIME	-Donation of money (1.8 M euros) for purchase of medical equipment like masks, monitors, mobile respirators, etc	Remote work	Yes/ Yes
BANKING	NATIONAL BANK OF GREECE	-Donation of respirators (11pcs) for adults, kids and babies	-Remote work	Yes/ Yes
TOURISM- HOTELS	SANI- IKOS	- Donation of medical equipment of 150 K euros in local hospitals - Customized internal Covid protocol & Covid Certificate from international certification & inspection agency	No info provided	Yes/ Yes
TRANSPORTATIONS	AEGEAN AIRLINES	- Free flights & transformation of airplanes for transportation of medical cargo - Free air tickets to health personnel	No info provided	Yes/ Yes
TELECOMS	COSMOTE (ΟΤΕ)	-Donation of respirators (25 pcs), beds and rest equipment for ICU (2 M euros) - Free extra GBs, SMSs and time for mobile phones - Limited free access to cable TV content	80% remote work	Yes/ Yes
TOBACCO	KARELIA TOBACCO COMPANY	Donation of 50 fully equipped ICU stations	No info provided	Yes/ Yes
ELECTRONICS INDUSTRY	INTRACOM HOLDINGS	Donation of 50 pressure respirators & other health equipment	No info provided	Yes/ Yes
OIL& GAS	HELLENIC PETROLEUM GROUP (ELPE)	Donation of -8 M euros including 2 Full Automated Molecular Diagnostic Stations (NeuMoDx 96) & health lab equipment - Donation of oil, food coupons, health protective equipment and antiseptic consumables to charity organizations	No info provided	Yes/ Yes
POWER GENERATION	PUBLIC POWER CORPORATION (DΕΗ)	-Donation of protective masks & uniforms (5 M euros) -Discount in customers due to increased home consumption (kWh discounted price)	-Remote work -Shift work -Minimum necessary personnel	Yes/ Yes
PUBLIC UTILITIES	HELLENIC POST (ΕLΤΑ)	Free transportation & distribution of medical supplies and personal protective equipment	-Remote work -Disinfection of workplaces	Yes/ Yes
SOAPS & DETERGENTS/ COMMODITY CHEMICALS	PAPOUTSANIS	-Donation of personal health & care products & medical equipment (defibrillator, etc.) -New exclusive vertical production line for biocides and alcohol disinfectants with stable low price	No info provided	Yes/ Yes
SUPER- MARKETS	AB VASILOPOULOS	Donation for purchase of masks for police & municipal authorities (0.5 M euros)	Bonus of 3 M euros to its employees of front line (stores & warehouses) with coupons	Yes/ Yes
HEAVY INSTUSTRY- METAL PROCESSING	MYTILINEOS	Donation of 65 respirators for ICU	-Remote work -Limitation of business trips to countries of high risk -PPE for coronavirus to personnel	Yes/ Yes
CONSTRUCTION	ELLACTOR	Provision of hazardous medical waste management services (transportation, incineration, et.) free of charge for a coronavirus hospital unit	-Remote work	Yes/ Yes
FOOD- BEVERAGES	COCA COLA HELLENIC BOTTLING COMPANY	-Donation of 3 ICU fully equipped -Reactors for 12.000 Covid tests - Help at Home program providing food and psychological support in vulnerable people - Future Loading program for the enhancement of small size HoReCa business during the pandemic with seminars for HSE certifications, recycling, sustainable development in cooperation with institutions and government	-Remote work -Electronic seminars for psychological support to employees -Electronic seminars for self-development -Internal guide for mindfulness and mental health	Yes/ Yes

These types of CSR actions have a few significant characteristics. There is a high pace regarding the planning and implementation phases which is due to the urgency of the pandemic. The top management and the shareholders of companies present decisiveness for taking actions. There is cooperation among firms, government, and National Organization of Public Health of Greece (EODΥ). A total net of stakeholders of the pandemic is fully activated and corporations have managed to establish communication, cooperation and coalitions with charity organizations, municipalities, civil protection organization, police, hospitals, respective ministries, etc. for common social initiatives. On parallel, there is everyday communication from firms’ side with their employees. Advices taken from specialists regarding manpower health protection against coronavirus prior of measurements taken by firms. Finally, corporations publish their CSR actions through media providing a good example of contribution to a global threat.

The total number of Covid-19 cases in Greece and the number of the available ICUs have been used by the researcher as key performance indicators for this study regarding the national effort to curb the pandemic, part of which consist the examined CSR actions of firms. The reaction of firms during this pandemic is prompt, specific, organized, effective, public, and measurable.

- It is prompt due to urgent situations that motivated and energized managers to act quickly.
- It is specific because the pandemic created a huge and specific need for enhancement of the National Health System capacity and the protection of employees. The target was too big to be lost.
- It is well organized because companies asked guidance and cooperation from the state and the National Organization of Public Health (EODY).
- It is effective because it is well organized and generous regarding its resources (staff and budget).
- It is public meaning that it is announced in public and presented through media in society. People and firms’ employees are interested to learn about it.
- Finally, all these actions are measurable signaling a total national positive performance against Covid-19 by examining the cases in working environment, in society in general, and the ICU availabilities.

This study recognizes methods, motives, axes of actions, good practices, common elements, etc. that could allow us to describe better a firm’s ability to react in such an unprecedented situation of a pandemic. It examines any new prospect that could arise after the adjustment of CSR policies to pandemic conditions and finally how much we could claim that this pandemic could be transformed into a chance for big corporations to realize deeper their corporate responsibility and transform themselves into corporate citizens.

## Proof development

The definitions of tactical and strategic CSR reveal the main parameters which are examined for this categorization. These are relevant to a) time, b) human resources commitment either regarding employees needed to plan and implement a CSR activity or regarding the number of employees involved in general, c) size of investment, d) correlation with core activities and e) impact on organization structure or on everyday activities of firm**.**

Indicatively strategic CSR implies: a) medium to long term regarding time, b) big human resources commitment either regarding employees needed to plan and implement a CSR activity or regarding the number of employees involved in general, c) medium to big size of investment, d) clear correlation with core activities and e) clear impact on organization structure or everyday activities of firm. Proportionally, tactical CSR implies: a) short to medium term regarding time, b) small to medium human resources commitment either regarding employees needed to plan and implement a CSR activity or regarding the number of employees involved in general, c) small to medium size of investment, d) no correlation with core activities and e) no impact on organization structure or everyday activities of firm.

The researcher has grouped the urgent CSR actions described in Table 1 into 12 categories presented in Table 2 (first column) and examines them from the point of view of these parameters. The results are colored either with light grey or dark grey depending on where they belong, in tactical or strategic CSR accordingly. There are in brief:

- 12 types of actions
- 5 parameters to be considered
- 60 boxes to be filled
- 24 boxes referred to strategic CSR and 36 boxes to tactical CSR
- 25% of types of actions are tactical and the rest 75% is a mix of tactical and strategic CSR

**Table 2:**
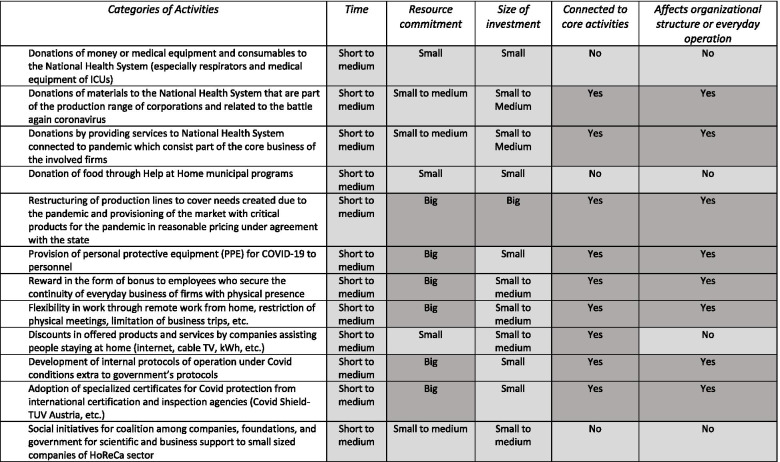
Analysis of urgent CSR actions due to COVID-19 through the prism of tactical (light grey) and strategic (dark grey) CSR parameters

We can see as a helicopter view from Table 2, that these random activities consist a mix with 40% of strategic content and 60% of tactical considering the boxes in total. Thus, we cannot categorize with certainty these activities due to the pandemic either as tactical or strategic since in general for a single type of action the characteristics are not solely tactical nor strategic. There are only three types having only tactical characteristics among 12 groups in total (only light grey boxes in row). The rest nine types (75%) combine characteristics from both tactical and strategic CSR. Therefore, it is reasonable to examine the possibility a new kind of CSR to have been created under these special unprecedented conditions of a dual health and economic crisis with a high uncertainty level about the scale and the duration of it. This new form of CSR appears as a hybrid between strategic and tactical CSR having common elements from both.

## Analysis of critical CSR

Knowing that the pandemic is an unscheduled and turbulent phenomenon with an unspecified duration, we could recognize a strong and very interesting contradiction. The current CSR policy of firms has been oriented to urgent goals, the previously designed short-terms targets have been changed, the tactical CSR has been reformed. The CSR reply to the current pandemic looks tactical since it is unexpected, flexible, short-term, and fast track. On the same time it looks strategic since it is robust regarding its resources and significance, and powerful concerning its possibilities for building trust and social capital (Sacconi & Degli Antoni, 2011). Thus, during this pandemic that is considered simultaneously a global recession, a new CSR approach has been raised combining characteristics of both tactical and strategical CSR. A window of threat and chance is come up and therefore urgent CSR programs are designed under fast track conditions which spring from a solid CSR strategy. This hybrid CSR activity maintains external characteristics which are tactical while its internal structural elements are strategic. We call this critical CSR since it consists a CSR configuration forged by the pandemic, the societal appeal for assistance from corporations and the governmental appeal for cooperation between public and private sector. This critical CSR is the answer to a great challenge which consists by a pandemic with unknown duration and an aftermath recession.

Herein, there is one extra contradiction. Firms’ contribution to national bids to curb COVID-19 initially seems to belong in the field of altruistic CSR as a charity action during a critical period of a pandemic. However, the fast response in the call from Greek government to private firms to support national efforts to furnish Greek National Health System is not just due to their charity reflexes. These firms are international companies, protagonists in their sectors and they work on CSR field for several years. They have realized the importance of the complex net of stakeholders, the need for sustainable characteristics in today’s business world and they have experienced the global financial crisis of 2007–2009 and the aftermath depression in Greece that longed eleven years. The precision of actions, the prompt response, the increased budget despite of the forthcoming recession and the range of actions reveal that there is a strategic thinking behind them. Finally, these urgent CSR actions are coming as products of strategic CSR rather than altruistic CSR, serving firms’ stakeholders and firm’s long- term goals.

The presented CSR programs are well planned, including measures of protection for employees, assistance to the National Health System and prototype actions like changes in company’s production lines. Such decisive actions do not allow ambiguities and are coming from clear managerial judgment. The researcher condenses all the above analysis in Table 3 providing the footprint of this novel CSR among the rest already recognized types. There are nine types of urgent CSR actions in Table 2 that are categorized as a hybrid between tactical and strategic CSR, thus as critical CSR. Furthermore, they belong to strategic CSR as per Carrol’s categorization and refer to an interactive firm (Table 3- fourth column). The rest three types of urgent CSR actions in Table 2 are tactical CSR, thus, they belong to altruistic CSR and refer to a responsive firm (Table 3- third column). Concluding, this novel form of CSR can be analyzed as per the already established theory and contributes in the further development of CSR theoretical background by highlighting new aspects of it.

**Table 3:** Theoretical profile of Critical CSR

THEORETICAL ANALYSIS OF CRITICAL CSR PROGRAMS DUE TO CORONAVIRUS			
**Type of Planning**	Tactical or Strategic	Tactical or Strategic	**Critical: Both Tactical & Strategic**
**Types of CSR** **(**Carroll, 1991**)**	Ethical	Altruistic	**Strategic**
**Type of Company** **(**Preston & Post, 2013; Sethi, 1979**)**	Reactive	Responsive	**Interactive**

Taking as fact the good work has been done by Greek companies, like the examined in this paper, during the pandemic, as the key performance indicators reveal, having the certainty that a financial downturn is coming aftermath, this could be interpreted as a proof of a well embedded CSR concept inside organizations’ culture and as a well-established understanding of corporate governance from top management (Bitektine, 2011). On parallel, this could be seen as a chance for an organization to build better relations with its stakeholders and enhance its branding (Mc Williams & Siegel, 2011).

## Conclusions

Crises are part of the capitalistic economic system. The combination of a health and financial threat creates unique turbulent conditions for the planet, its citizens, and corporations. Several firms worldwide have expressed their interest for COVID-19 confrontation either to the direction to protect their employees or to support community beyond the measures taken by the states. Most of international companies have used the possibilities for remote work from home for their employees. Moreover, there have taken place some novel and radical actions like the car industry SEAT in Spain which has transformed partially its productions lines in order to assist by manufacturing ventilators for hospitals (From Making Cars to Ventilators, 2020). Thus, there is a similarity among the urgent CSR by firms worldwide with those described in the present study for Greece.

So, we infer that the CSR policies developed internationally during the pandemic are tactical and strategical on the same time. Although these CSR actions seems to be developed in the field of altruistic CSR, they spring from strategic CSR field. This phenomenon reveals to us a new universal form of CSR, called critical CSR which comes up as a reaction to a dual health and financial global crisis. Furthermore, we conclude that modern companies have shifted from shareholder’s theory for the maximization of profit (Friedman, 1970) to stakeholder’s theory (Carroll, 1991). Since we are currently within the health and financial crisis due to COVID-19 the academic work about its implications to CSR activities of firms is scarce. However, a lot of research has been done for the financial crisis of 2007–2009 and how this has affected CSR. This pandemic consists a very interesting challenge to examine a more complicated phenomenon with a dual crisis in health and financial level, and a novel universal category of CSR. Critical CSR does not cancel the classification between tactical and strategic CSR. On the contrary critical CSR covers the gap between these two forms, enriches the theoretical background of CSR in general and provides a better explanation for firms’ urgent CSR actions to fight the pandemic.

CSR programs of international Greek firms have flourished during the pandemic as a reaction to increased needs for employees’ protection from COVID-19 and for National Health System support. The enhanced CSR activity sends a clear message that CSR during crises could resist and even get stronger under certain management. The successful reaction of firms in the Greek case provides the opportunity to managers to investigate the attributes of these successful CSR programs that could enrich their usual scheduled CSR policy.

Modern firms should listen the needs and concerns of their internal and external environment. They should adjust their CSR strategy on the extracts of the communication with their stakeholders. They should be adapted to new conditions and adjust their CSR policy accordingly. When a CSR policy comes because of such interaction is going to be to the point, modern and useful. The firm could have the possibility to direct its available resources into the correct direction and put specific targets to be achieved. Having these parameters fulfilled the CSP of the organization would be high and this should be measured. The possible advantages from CSR actions could return into the firm through its branding, employees’ royalty, consumers and employees’ satisfaction and approval. Finally, the pandemic could teach that firms could be valuable corporate citizens and they have the power to be part of the solutions of the problems of our world.

Moreover, there have been companies without established CSR policy which have implemented critical CSR actions during this pandemic, and this is a very interesting element for further investigation whether this experience of urgent CSR actions could lead them to re- examine their position in relation with CSR. Urgent but well-organized CSR programs in the frame of confrontation of the pandemic for both firm’s employees and local community as these described for Greek companies could not be considered superficially as an opportunistic behavior by firms. Even though this specific CSR policy could target to accrue benefits for an organization, it relates to important elements of business administration like stakeholders’ trust, social capital, organizational identification, CSP, civic engagement and branding. Moreover critical CSR policy during the coronavirus period reveals important information about company’s flexibility to adjust to turbulent conditions, its risk management capability to invest in CSR in the threshold of a global recession, its ability to reform CSR policy directly in order to catch up pandemic high pace of development and its willingness to support community. Finally, we could conclude that this pandemic could be considered as a chance for organizations, especially those with moderate CSR performance, to invest in critical CSR programs and increase their resilience to the forthcoming recession ex ante rather than ex post.

## Data Availability

All data used for this article are already publicly available. The list of internet addresses indicates the source of information about modern CSR of firms during the pandemic. Moreover, there are not individual person’s data in this article.

## Abbreviations

CFP: Corporate Financial Performance
CSP: Corporate Social Performance
CSR: Corporate Social Responsibility
HoReCa: Hotel- Restaurant- Cafe
ICU: Intensive Care Unit
OECD: Organization of Economic Cooperation and Development
PPE: Personal Protective Equipment
